# Timing of Splenectomy after Acute Spinal Cord Injury

**DOI:** 10.1523/ENEURO.0440-21.2021

**Published:** 2022-01-25

**Authors:** Feng Wu, Xiao-Hui Li, Min-Jie Gong, Jia-Qi An, Xiao-Yan Ding, Sheng-Li Huang

**Affiliations:** 1 Center of Teaching and Experiment for Medical Post Graduates, School of Medicine, Xi'an Jiaotong University, Xi'an 710061, China; 2 Departments of Radiology, The Second Affiliated Hospital, School of Medicine, Xi'an Jiaotong University, Xi'an 710004, China; 3 Departments of Otorhinolaryngology, The Second Affiliated Hospital, School of Medicine, Xi'an Jiaotong University, Xi'an 710004, China; 4 Department of Neurology, The First Affiliated Hospital, School of Medicine, Xi'an Jiaotong University, Xi'an 710061, China; 5 Department of Ophthalmology, Xi'an No 3 Hospital, Xi'an 710021, China; 6 Department of Orthopaedics, The Second Affiliated Hospital, School of Medicine, Xi'an Jiaotong University, Xi'an 710004, China

**Keywords:** acute, spinal cord injury, splenectomy, timing

## Abstract

Spinal cord injury (SCI) is a devastating condition. Splenectomy may play a protective role in the development of SCI. However, little is known about whether the timing of splenectomy affects the outcome after SCI. Investigation into splenectomy after SCI would provide insight into how the timing can be selected following SCI to improve neurologic outcomes. Rats were randomized into a sham group, a nonsplenectomized group (NonSPX), four splenectomized groups with the surgery performed immediately, 6 h, 12 h, and 24 h after SCI (SPX0, SPX6, SPX12, and SPX24, respectively). Rats were subjected to severe contusive SCI at the level of the third thoracic vertebra. At different time points following SCI, Basso, Beattie, and Bresnahan (BBB) score was used to assess the recovery of injury. The animals in each group were randomly selected for tissue collection at days 3, 14, and 28 after surgery. Then, immunohistochemistry of immunologic cells was performed and inflammatory mediators were determined. Our study showed that splenectomy within 6 h after SCI improved BBB scores as compared with splenectomy more than 12 h after SCI, and decrease the immune cell responses to SCI. Protein levels of interleukin (IL)-1β and tumor necrosis factor (TNF)-α were significantly elevated in nonsplenectomized group compared with sham group. No difference was observed in IL-10 at the lesion site between splenectomized and nonsplenectomized groups at 3 d post-SCI. The study demonstrates that splenectomy within 6 h after SCI would lessen the development of SCI and improve outcome.

## Significance Statement

The pathophysiologic changes in spinal cord injury (SCI) involve secondary injury from the immune and inflammation system. The spleen is the largest immune organ in the mammalian lymphatic system. In animals, both splenectomy before or immediately after brain injury can alleviate the acute brain injury. However, splenectomy after SCI has not been extensively studied. Our study indicates that the splenectomy within 6 h after SCI limits neuroinflammatory pathology and improves neurologic recovery. Our results implicate splenectomy could prove to be a therapeutic option to reduce injury in modulating the immune response following SCI. Therefore, the present study may form a novel strategy for biological and clinical research in SCI in the future.

## Introduction

Spinal cord injury (SCI) is a devastating condition in which motor and sensory impairments hamper the patients’ quality of life. Given the great efforts by medical researchers and health care workers, it remains a major challenge today (Huang et al., 2014, 2018; Li et al., 2015, 2017; Liu et al., 2017, 2022; Moritz, 2018). The pathophysiologic mechanisms of the injury have not yet been clearly elucidated. A better understanding of the pathophysiology would be a critical step toward improving or renovating the therapeutic strategies in management. As is already proved, inflammatory cells and inflammatory agents are released immediately after SCI and result in secondary damage to the injured cord tissues (Zong et al., 2012; Boato et al., 2013; Brommer et al., 2016; Mukhamedshina et al., 2017). This suggests that the immune and inflammatory reaction is an important factor for SCI.

The spleen, the largest immune organ, serves as a reservoir for immune cells (Offner et al., 2006; Wei et al., 2006). It contains a wide variety of immune cell populations, including B and T lymphocytes, and monocytes. Several studies have demonstrated that the spleen can cause secondary damage to the spinal cord after SCI through the release of proinflammatory cytokines such as interleukin-1 β (IL-1β) and tumor necrosis factor-α (TNF-α; Koj, 1996; Pomeshchik et al., 2015). These inflammatory cytokines eventually enhance the posttraumatic immunologic response. In addition, our previous work revealed that the spleen undergoes profound atrophy after higher level SCI, and SCI can potentiate inflammatory responses and the spleen can release inflammatory cells (Wu et al., 2019, 2020).

Interestingly, previous studies have shown that surgical removal of the spleen (splenectomy) before or immediately after brain injury plays a protective role in a variety of brain injury models (Li et al., 2011; Ostrowski et al., 2012; Dotson et al., 2015; Pennypacker and Offner, 2015). The spleen is incriminated for exacerbating acute brain injury at the early stage. Recently, splenectomy is reported to be able to improve the outcome in SCI rats (Blomster et al., 2013; Temiz et al., 2013). These studies suggest that inhibition of splenic function before or immediately after SCI is neuroprotective and significantly reduces inflammation early after SCI. Therefore, splenectomy may possibly hinder or relieve the immune and inflammatory reaction secondary to SCI.

Splenectomy has been reported to be performed at different time points in SCI cases. However, there are no data comparing the outcomes in terms of the timing of splenectomy (the optimal time for splenectomy). It is not clear whether this timing would impact the effect of splenectomy on SCI, but it would be of interest to examine whether a relation exists between the timing of splenectomy and the recovery after SCI. Trying to gain an understanding, the present study evaluated the timing of splenectomy after acute SCI on long-term outcomes.

## Materials and Methods

Adult male Sprague Dawley rats, weighing 220–260 g, were obtained from the Experimental Animal Center of Xi'an Jiaotong University. The study was approved by the Animal Experiment Committee of the university. All experimental procedures were conducted in accordance with the guidelines established by the National Institutes of Health (NIH). Before the surgical procedure, rats were kept in cages for one week under a 12/12 h light/dark cycle and with unlimited access to food and water.

### Materials and reagents

SCI impactor NYU was obtained from a weight-drop device, New York University (New York, NY). Mouse anti-rat CD3 (catalog #GTX76493 no. 821200995) was obtained from GeneTex Inc. Mouse anti-rat MPO (catalog #sc-390109 no. C2018), mouse anti-rat CD45RA (catalog #sc-53 048 no. H2917), mouse anti-rat IL-1 (catalog #sc-52012 no. D2418), mouse anti-rat TNF-α (catalog #sc-52746 no. D0518), and mouse anti-rat IL-10 (catalog #sc-365858 no. K1617) were purchased from Santa Cruz Biotechnology. Mouse anti-rat ED1 (catalog #ab31630 no. GR278101-19), mouse anti-rat Glial fibrillary acidic protein (GFAP; catalog #ab7260 no. GR138562-1) and mouse anti-rat neurofilament protein 200 (NF200; catalog #8259 no. GR190-41) were purchased from Abcam. Horseradish peroxidase (HRP)-conjugated anti-mouse was purchased from BOSTER Biological Technology. Diaminobenzidine were purchased from MXB Biotechnology.

### Animal grouping

A total number of 120 Sprague Dawley rats were randomly divided into six groups (*n* = 20): sham (laminectomy plus laparatomy, without SCI and splenectomy), NonSPX (SCI plus laparatomy, without splenectomy), SPX0 (splenectomy immediately after SCI), SPX6 (splenectomy 6 h after SCI), SPX12 (splenectomy 12 h after SCI), and SPX24 (splenectomy 24 h after SCI). NonSPX was called nonsplenectomized group, and SPX0, SPX6, SPX12, and SPX24 were called generically splenectomized groups. After surgery, five animals in each group were randomly selected for tissue collection at days 3, 14, and 28, and another five were randomly selected for protein extraction from the spinal cord at day 3. After surgery, an antibiotic agent (Cefazolin) was administered daily by intramuscular injection for 3 d. All animals were given a subcutaneous injection of buprenorphine (0.05 mg/kg) twice a day for 2 d after surgery.

### Spinal cord contusion procedure

The detailed experimental procedure was described in our previous study (Wu et al., 2019). In brief, the rats were anesthetized with an intraperitoneal injection of pentobarbital at a dose of 30 mg/kg body weight. A laminectomy was performed at vertebral level T3 to expose the dorsal spinal cord, and then a spinal contusion was made by the SCI impactor, with a 10-g rod dropping from a height of 50 mm. After the treatment, rats were housed in individual cages and had free access to food and water. After SCI, bladders were manually expressed twice daily for the duration of the experiment. The sham group only underwent T3 laminectomy, without SCI.

### Splenectomy

Splenectomy was performed immediately or at 6, 12, or 24 h after SCI induction. The skin at the level of the thirteenth rib was shaved and sterilized. A skin incision of ∼2 cm was performed at the level of the left costal margin, through which the spleen was exteriorized with blunt forceps. The splenic artery, veins, and nerves were ligated and the spleen removed. The abdominal wall and incision were then closed with sutures. For the sham and nonsplenectomized groups, laparatomy was performed without removing the spleen. The spleen was exteriorized for 2 min and reinserted into the cavity. Then, the abdominal wall was closed with sutures.

### Behavioral analysis

Before collection of the spinal cord samples, post-SCI functional outcomes were evaluated by Basso, Beattie, and Bresnahan (BBB) scoring system (Basso et al., 1995, 1996). The evaluation was performed by two independent and blinded observers every 3 d after the surgery until the animals were killed.

### Magnetic resonance imaging (MRI) and diffusion tensor imaging (DTI) scans

Conventional MRI and DTI scans were conducted *in vivo* at days 3, 14, and 28 after the surgical treatment. MRI acquisition and DTI data analysis were performed as described previously (Li et al., 2015, 2017). Briefly, all MRI examinations were performed on a 3.0 Tesla MR Scanner (Signa, GE Medical Systems) followed in configuring dedicated scanner at 3 Tesla for studying rat models. Conventional MRI scan, including T1-weighted and T2-weighted images (T1WI and T2WI) was completed with the SE sequence. Sagittal T2WI was acquired using the following parameters: TR/TE of 2200 ms/126 ms, image matrix of 256 × 256, and six contiguous slices, with a slice thickness of 1.5 mm. The parameters for sagittal T1WI were: TR/TE of 440 ms/11.1 ms, image matrix of 256 × 256, and six contiguous slices with a slice thickness of 1.5 mm. DTI was acquired with identical geometry as the anatomic images using single shotspin-echoplanar imaging (EPI) sequence with TR/TE of 4000 ms/88 ms, slice thickness of 3 mm, b factor of 1000 s/mm^2^, bandwidth of 200 kHz, 25 gradient encoding directions, acquisition matrix of 64 × 64, and field of view 10 × 10 mm. After image acquisition, the data were transferred to an independent workstation to calculate the DTI indices. Diffusion tensor tractography (DTT) of the spinal cord was generated using the FACT algorithm implemented in Volume-One software, and fractional anisotropy (FA) threshold <0.2 and stopping angle of >25° were used as parameters.

### Immunohistochemistry

Rats were deeply anaesthetized with an intraperitoneal injection of pentobarbital, followed by transcardial perfusion with ice-cold PBS to remove blood till the lung turned white, and then with freshly hydrolyzed paraformaldehyde (4%) in 0.1 mol/l phosphate buffer at pH 7.4. The spinal cords were then harvested, postfixed in the same fixative for 24 h. A 2-cm segment of spinal cord at the contusion zone was embedded in paraffin, and then sectioned transversely one sliding microtome at a thickness of 5 μm.

For immunohistochemistry, the tissue sections were deparaffinized in xylene and rehydrated. The sections were treated with 3% hydrogen peroxide for 20 min to block endogenous peroxidase activity, and then placed in 0.1 mol/l citric acid buffer solution (pH 7.42) and kept in microwave at 92–98°C for 13 min for antigen retrieval. After cooling at room temperature, the sections were washed with PBS three times for 5 min each time, and incubated with 10% goat serum for 15 min. Next, for primary antibodies preparation used in this experiment including: mouse anti-rat GFAP (1:1000), mouse anti-rat NF200 (1:200), mouse anti-rat CD3 (1:50, CD3 for T cell), mouse anti-rat CD45RA (1:200, CD45RA for B cell), mouse anti-rat ED1 (1:1000, ED1 for macrophage), and mouse anti-rat MPO (1:200, MPO for neutrophil). The sections were incubated with one of the primary antibodies overnight at 4°C in a humidified chamber. After that, the sections were washed with PBS three times, incubated with goat HRP-conjugated anti-mouse for 30 min at 37°C, and again washed with PBS three times. Then HRP was detected with diaminobenzidine. The sections were counterstained with hematoxylin, dehydrated, and mounted. Negative controls were processed according to the same protocol but the primary antibodies were omitted.

Histologic information was used to confirm the pathologic changes at the lesioned spinal cord. The number of positive cells was counted using the ‘count’ function using Image-Pro Plus (US Media Cybernetics; Sun et al., 2017), while immunostaining intensity of GFAP and NF200 obtained from the three different regions in each tissue were averaged to represent the specified marker (Li et al., 2017), and the average was taken as the measured value.

### Protein extraction from the spinal cord

Five rats from each group were decapitated 3 d after SCI. Spinal tissue within 2 mm to the trauma edge was harvested and stored at −80°C. Spinal cord tissues were homogenized in a precooled RIPA buffer by ultrasonic. The homogenate was incubated on ice for 30 min and centrifuged at 12,000 × *g* for 15 min at −4°C to collect supernatant. The protein content was determined using a bicinchoninic acid method (Beyotime Biotechnology Co).

### Immunoblotting analysis

A total of 50 μg of protein from each group was mixed with 5× SDS sample buffer. Equal amounts of protein were loaded onto polyacrylamide gels and then separated by electrophoresis at 100 V for 120 min. Proteins were transferred to a polyvinylidene difluoride (PVDF) membrane (Invitrogen) using the Bio-Rad Gel Transfer Device (Bio-Rad Laboratories) for 30 min. Following the transfer, the membranes were blocked in 5% nonfat dry milk in TBS containing 0.05% Tween 20 for 1 h. Monoclonal anti-rat IL-1β, TNF-α, and IL-10 were incubated with immunoblotting membranes at a dilution of 1:500 in 5% milk at 4°C overnight. On the following day, the membranes were washed three times with TBS and probed with a HRP-conjugated secondary antibody at a dilution of 1:5000 in 5% milk for 1 h, followed by TBS washing. Then, immunoreactivity was detected using an enhanced chemiluminescence kit (Millipore Corporation). The blots were normalized to β-actin (1:5000). Band intensity was quantified by NIH ImageJ software.

### Statistical analysis

Statistical analyses were performed using SPSS21.0 software. Data are presented as mean ± SEM. Significance of the data were determined by two-way analysis of variance with a Dunnet’s *post hoc* test for BBB scores. Two-tail independent *t* test was used to test for significant changes in FA values and histology parameters between groups. Differences with a value of *p* < 0.05 were considered statistically significant.

## Results

### Behavioral analysis

**Figure 1.**
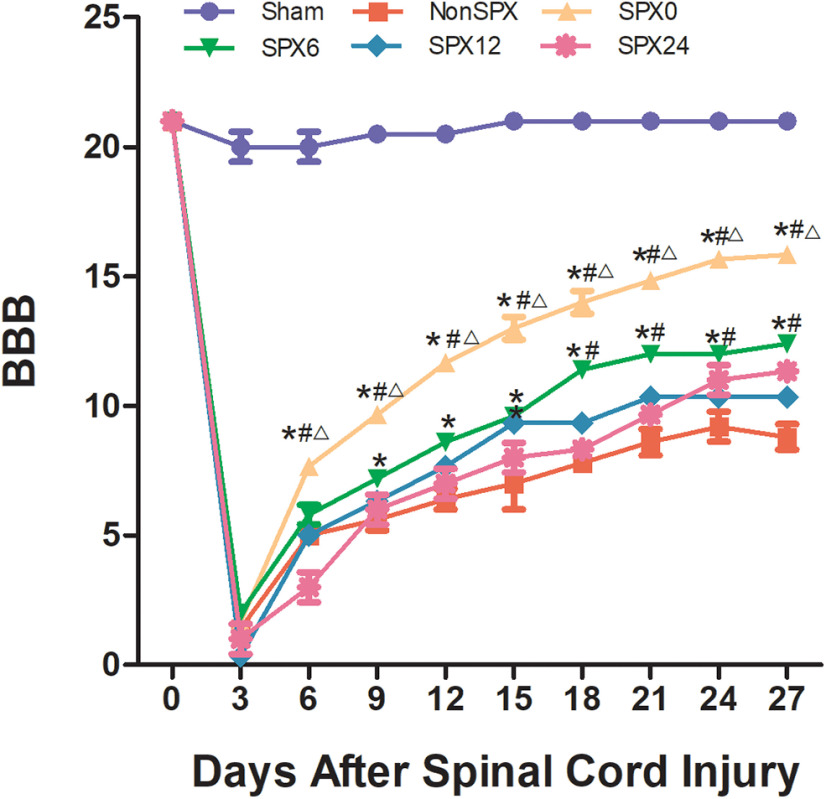
Behavioral analysis after SCI from each group at designed time points. BBB scores were significantly higher in SPX0 and SPX6 groups at day 9 and later versus NonSPX group, but there was no significant difference in BBB between splenectomy >12 h after SCI (SPX12, SPX24) and nonsplenectomized animals (NonSPX). *Compared with NonSPX group, *p* < 0.05. ^#^Compared with SPX12 group, *p* < 0.05. ^△^SPX0 compared with SPX6 group, *p* < 0.05.

The BBB score was significantly reduced on day 3 after SCI in the nonsplenectomized and splenectomized groups as compared with in the sham group, but the two groups with splenectomy over 12 h after SCI showed no improvement compared with the nonsplenectomized group at later time points (Fig. 1). Rats subjected to splenectomy within 6 h after SCI have increased BBB scores on day 9 and later as compared with splenectomy >12 h after SCI animals. Interestingly, BBB scores were significantly higher in SPX0 rats compared with rats in SPX6 at 6 and 9 d postinjury and thereafter until the experimental endpoint. These data suggested that splenectomy within 6 h after SCI might result in an improvement in long-term functional performance. The comparison of BBB scores revealed differences in recovery of locomotor function in different treatment groups, indicating that the timing of splenectomy did change SCI outcomes.

### MRI

**Figure 2.**
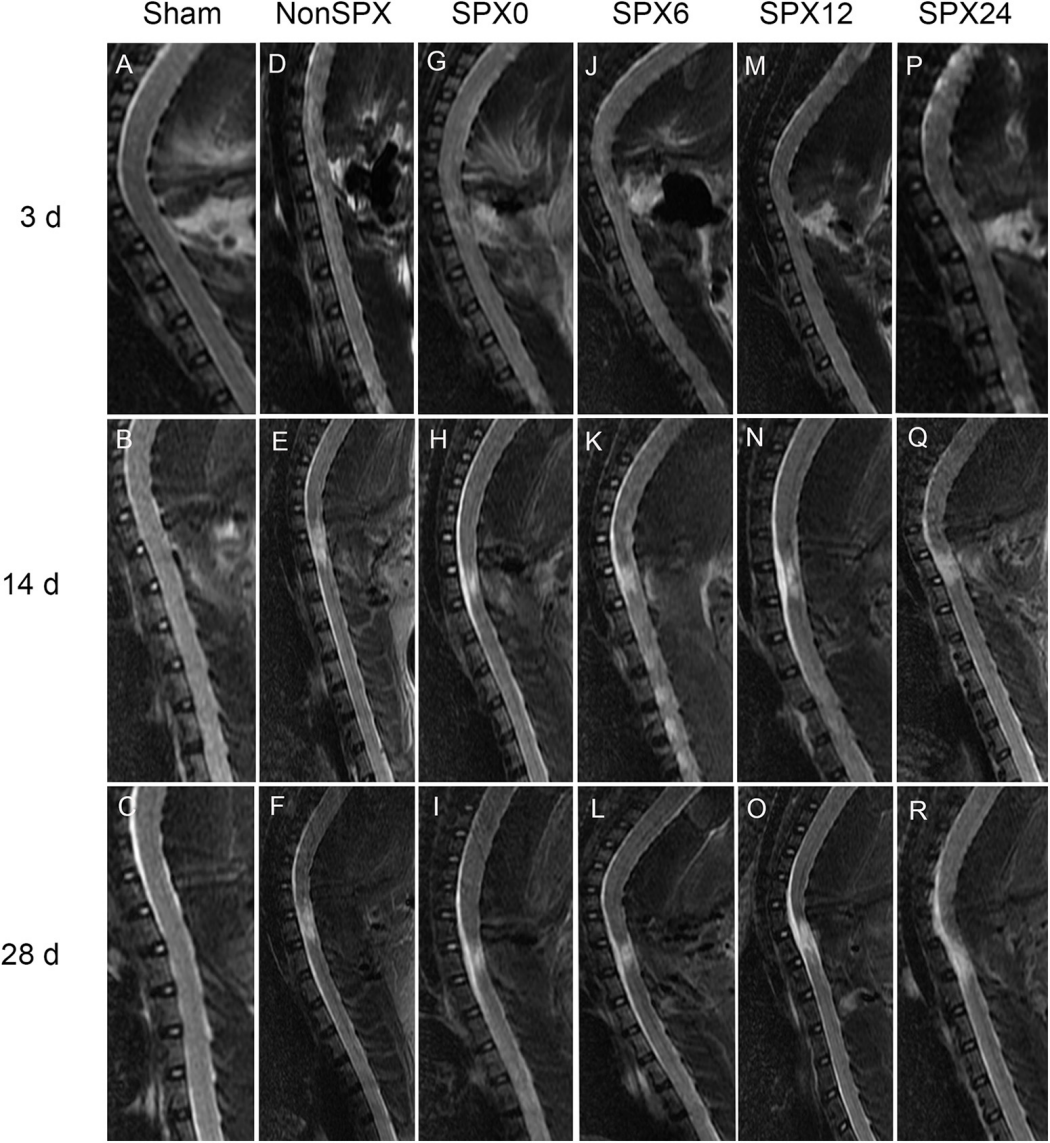
Conventional magnetic resonance T2-weighted images from each group at designed time points.

Spinal cords in the sham group presented normal morphology (Fig. 2). T2WI was used to assess the impact of splenectomy as it is more sensitive than T1WI for detecting alterations caused by SCI. At day 3, conventional midline sagittal T2WI in the splenectomized and nonsplenectomized groups demonstrated focal decreased signal of the spinal cord with surrounding abnormal increased signal intensity corresponding to the site of contusion. At days 14 and 28, circumambient hyperintense regions extended in both rostral and caudal directions from the injury epicenter in the plenectomized and nonsplenectomized groups.

### FA

**Figure 3.**
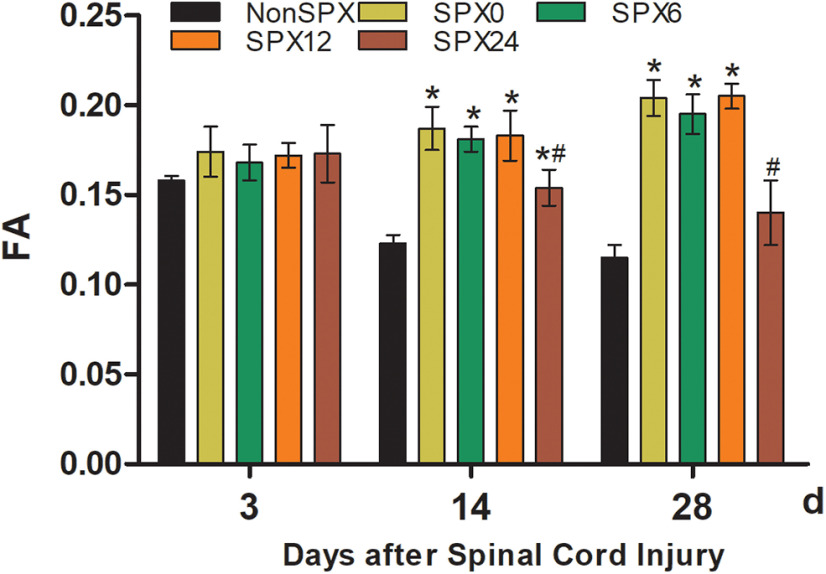
FA values from each group at designed time points. There was no significant difference between the plenectomized (SPX0 SPX6, SPX12, and SPX24) and nonsplenectomized (NonSPX) groups at 3 d after SCI. *Compared with NonSPX group, *p* < 0.05. ^#^Compared with SPX0 group, *p* < 0.05.

Conventional MRI revealed limited information about the state of SCI, but FA, one of the DTI parameters, is a sensitive biological marker for assessing the severity of structural damage of SCI, because there were different patterns of changes in FA value in terms of different injury severities and time points in our previous study (Li et al., 2017). At day 3 after SCI, the differences among FA values in the splenectomized and nonsplenectomized groups were not statistically significant (*p* > 0.05; Fig. 3). At day 14, the FA values increased in the splenectomized groups as compared with in the nonsplenectomized group (*p* < 0.05), especially the splenectomy within 12 h after SCI. Then, 28 d after SCI, there was no significant difference between the SPX24 group and the nonsplenectomized group. Surprisingly, the FA values decreased in the SPX24 group compared with the SPX0 group at days 14 and 28 after SCI (*p* < 0.05).

### DTT

**Figure 4.**
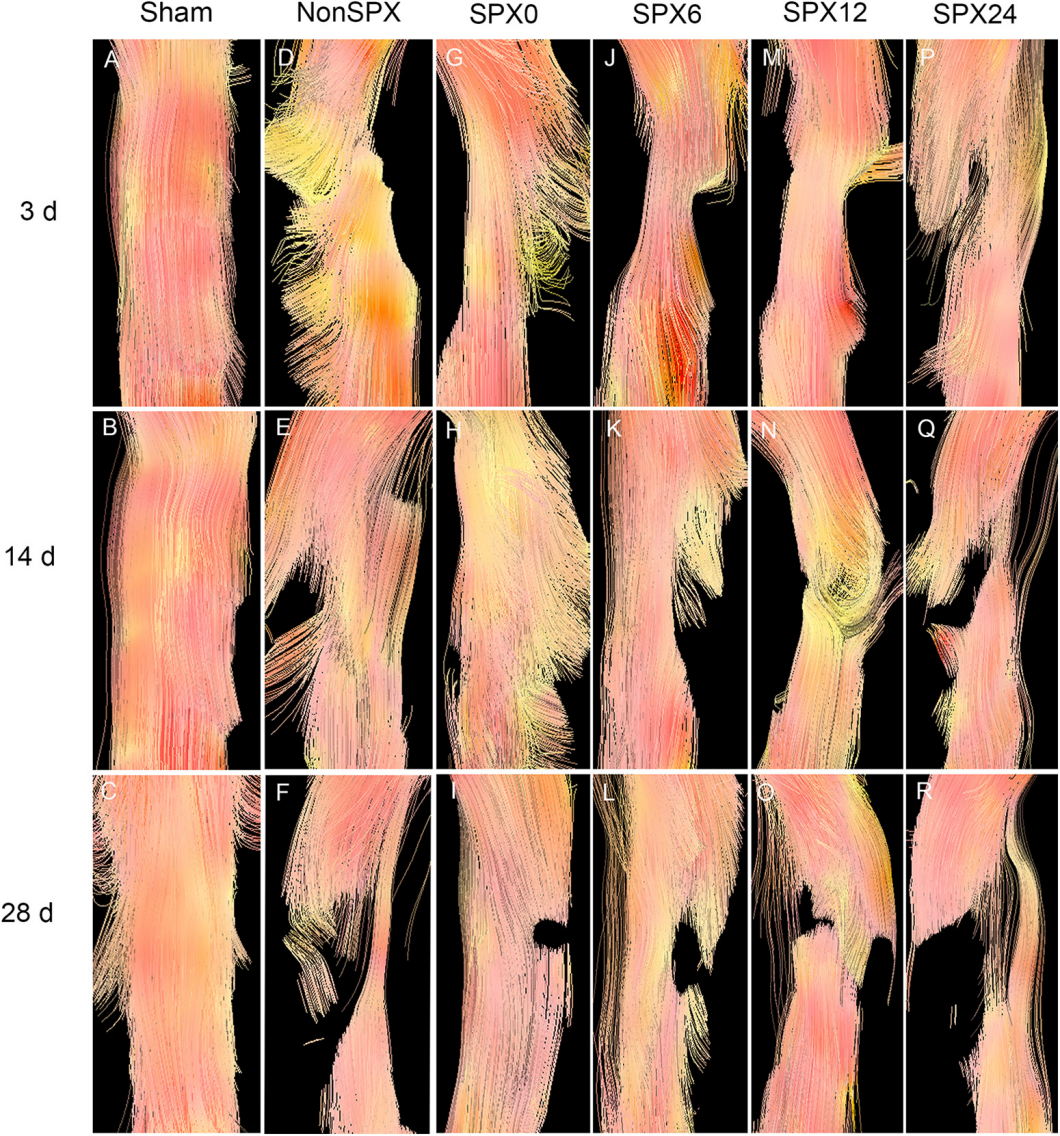
DTT in the spinal cord from each group.

The sham rats presented well-organized fiber tracking of the spinal cord tracts, while the rats in the splenectomized and nonsplenectomized groups showed irregularity in nerve fiber tracks (Fig. 4). At days 14 and 28, DTT in the rats receiving splenectomy over 12 h after SCI clearly demonstrated the lack of continuity of nerve fiber tracking, while that in the rats receiving splenectomy within 6 h showed irregularity in nerve fiber tracks, with the lack of continuity of some nerve fiber tracking. The spinal cord tissue of both splenectomized and nonsplenectomized groups did not show obvious changes comparing 14 and 28 d.

### Immunohistochemistry change

#### Glial fibrillary acidic protein

**Figure 5.**
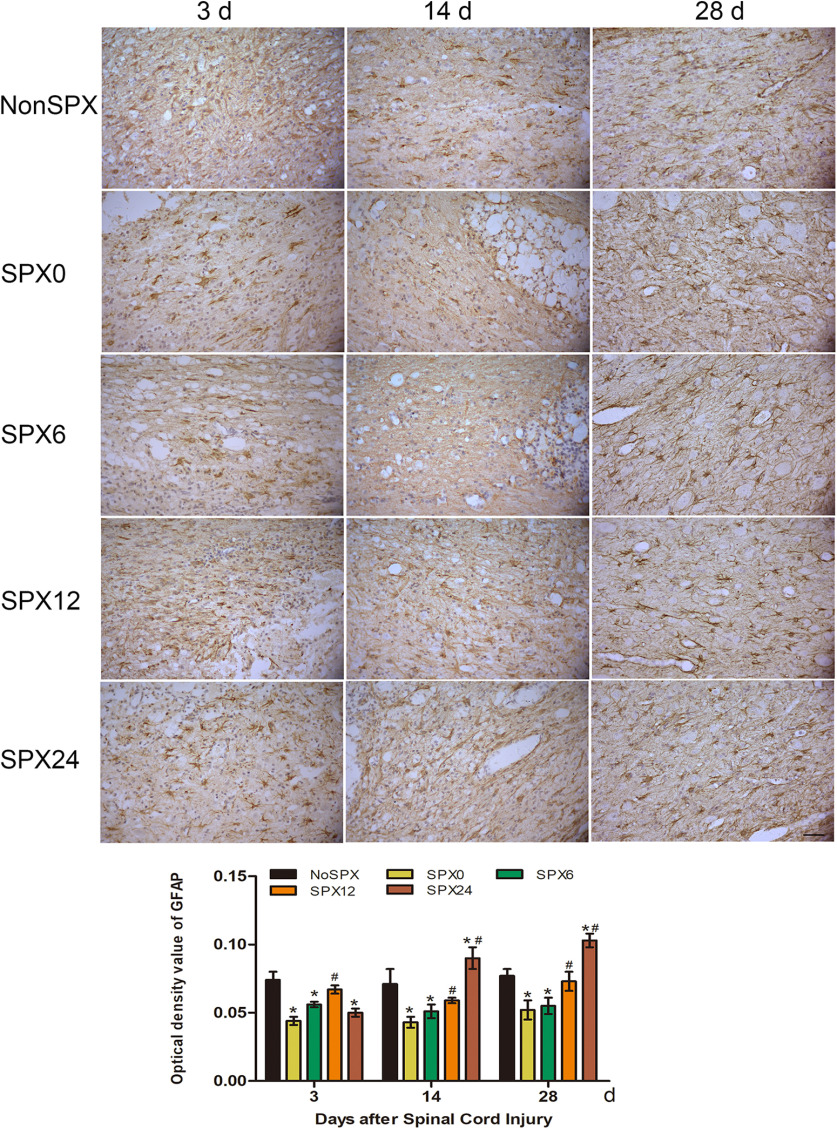
Immunohistochemical change of GFAP in the injured cord after SCI from each group (×400; scale bar: 50 μm). Expression of GFAP was lower in the splenectomy within 6 h. *Compared with NonSPX group, *p *<* *0.05. ^#^Compared with SPX6 group, *p *<* *0.05.

Spine cord tissue sections were immunostained for GFAP to investigate the recruitment of astrocytes in the injured spinal cord. In the nonsplenectomized group, the glial cells were more concentrated in the white matter of the spinal cord at day 14 than at day 3 (Fig. 5). In the splenectomy within 6 h, a small number of glial cells with hyperchromatic nuclei were present at day 3, and concentrated glial cells were not prominent at days 14 and 28 (Fig. 5). Although the expression of GFAP at days14 and 28 was lower in the splenectomy within 6 h than in the splenectomy >12 h, expression of GFAP also increased in time-matched splenectomy rats following SCI.

#### Neurofilament protein 200

**Figure 6.**
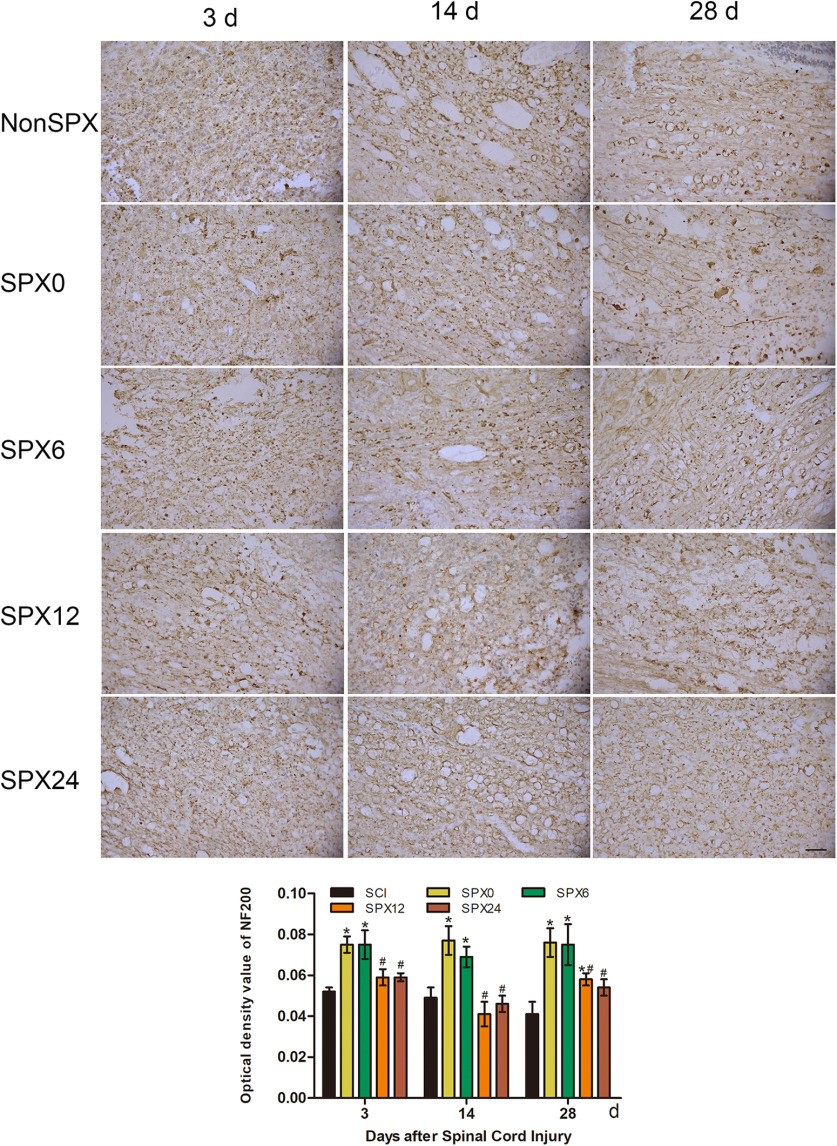
Immunohistochemical change of NF200 in the injured cord after SCI from each group (400×; scale bar: 50 μm). Acute splenectomy after SCI increased NF200. Expression of NF200 was higher in the splenectomy within 6 h. *Compared with NonSPX group, *p *<* *0.05. ^#^Compared with SPX6 group, *p *<* *0.05.

A decrease of neurofilament protein NF200, the main component of the framework of the neuron and axon, was observed in the nonsplenectomized and splenectomized groups (Fig. 6). At SCI, the difference between the splenectomy within 6 h and splenectomy >12 h was significant (Fig. 6). At days 3 and 14, there was a decrease trend in NF200 for splenectomy >12 h, although this trend was not more pronounced in splenectomy within 6 h after SCI.

### Cell inflammatory responses

**Figure 7.**
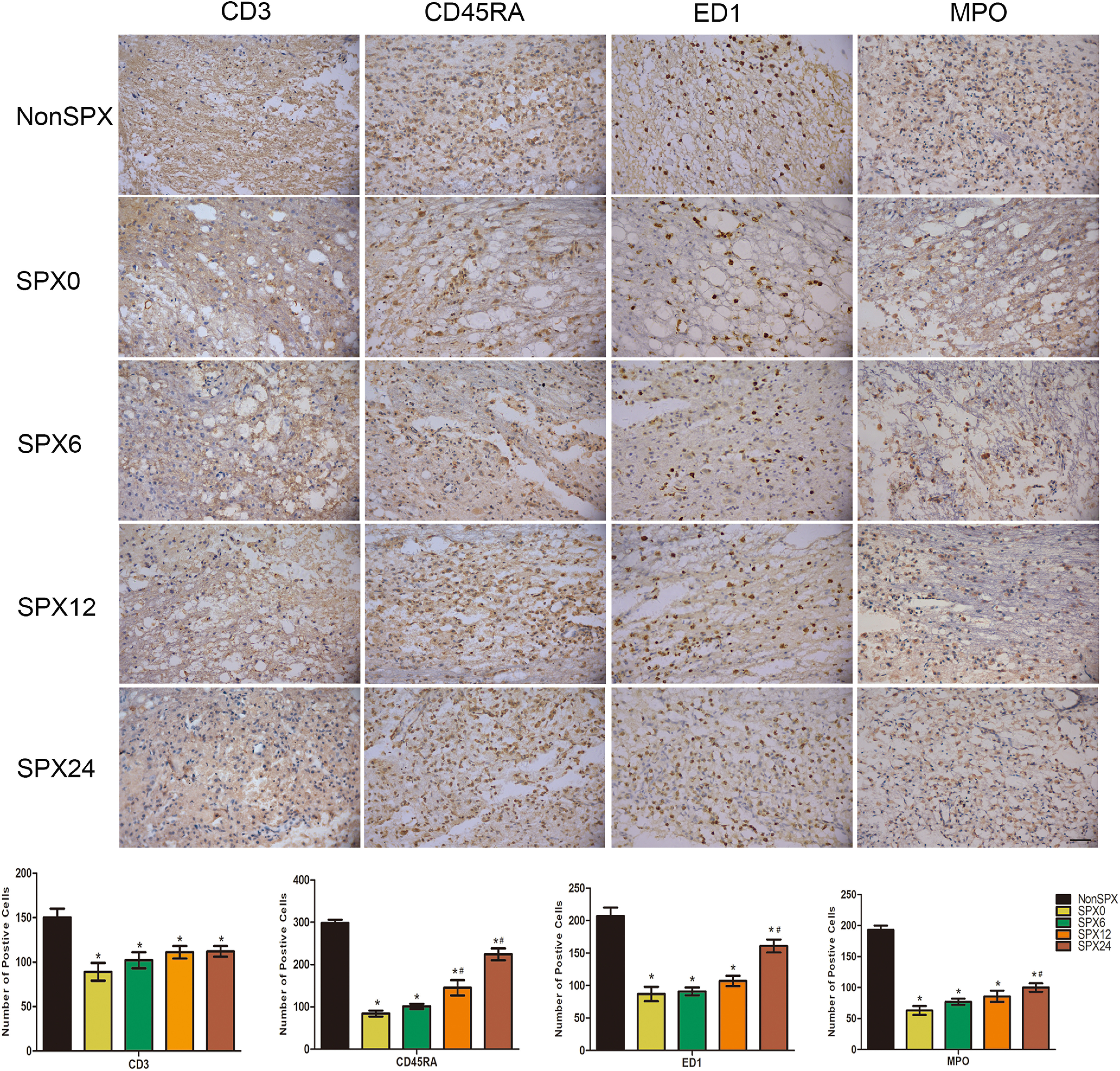
Peripheral immune cell infiltration into the injured cord on day 3 after SCI from each group (400×; scale bar: 50 μm). Immune and inflammatory cells in the injured cord decreased in splenectomized groups. Acute splenectomy relieved the inflammatory responses, although the increasing trend was more obvious in the SPX24 group. *Compared with NonSPX group, *p* < 0.05. ^#^Compared with SPX6 group, *p *<* *0.05.

To analyze the recruitment of immune and inflammatory cells in the injured cord, the infiltrating cells in the lesion epicenter were visualized at day 3 after SCI (Fig. 7), because inflammatory responses occur in the early stage of injury. The number of B cells, T cells, neutrophil and macrophages within the injured cord significantly decreased in splenectomized groups than nonsplenectomized (Fig. 7). There was significant difference between the SPX24 group and the splenectomy within 12 h. Especially CD45RA for B cell, the difference between the splenectomy within 6 h and splenectomy >12 h was significant. These results indicated that splenectomy within 6 h after SCI relieved the inflammatory responses of injured cords.

### Inflammatory mediators

**Figure 8.**
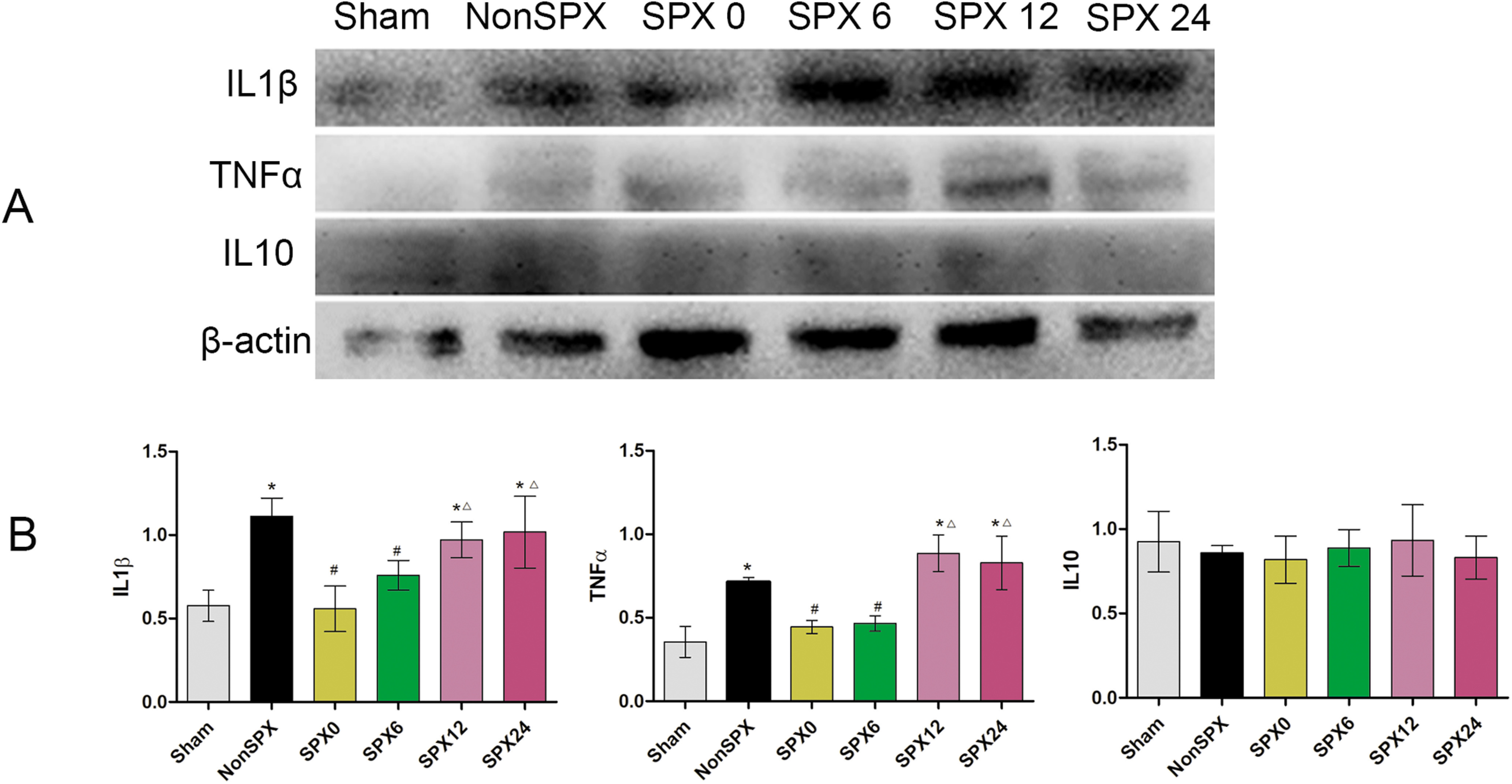
Inflammatory mediators expression in the injured cord after SCI from each group. ***A***, Immunoblotting analysis of protein extracts from day 3 after SCI in each group. ***B***, Quantified densitometric data of protein expression by statistical analysis. The levels of IL-1β, TNF-α, and IL-10 on day 3 showed the above results. *Compared with sham group, *p* < 0.05. ^#^Compared with NonSPX group, *p* < 0.05. ^△^Compared with SPX0 group, *p* < 0.05.

Splenectomy relates to inflammatory responses of injured cords, including not only inflammatory cells but also inflammation mediators, such as proinflammatory agents IL-1β, TNF-α, and anti-inflammatory cytokines IL-10. Our examination at day 3 following SCI uncovered a significantly greater presence of IL-1β and TNF-α in the injured cord tissue in the nonsplenectomized group than in the sham group (Fig. 8). Splenectomy within 6 h after SCI resulted in a dramatic decline in TNF-α and IL-1β present at the injury site compared with that in the nonsplenectomized group. These differences were, however, not detectable in the groups with splenectomy over 12 h after SCI. The level of IL-10 in the splenectomized groups did fall slightly in relation to their nonsplenectomized counterparts, but were not significantly different from sham and nonsplenectomized groups at set time points investigated (*p* > 0.05).

## Discussion

The present study investigated the effect of timing of splenectomy at the early stage of SCI on the long-term neurofunctional performance. The study differs from previous studies, as splenectomy was performed different time point after SCI. The comparative research design allows a unique investigation into the effect of timing of splenectomy on the neurologic outcome after SCI. Our experiments demonstrated that splenectomy within 6 h after SCI decreased the infiltration of immune cells into the injured spinal cord, proinflammatory cytokines, and attenuate immune responses to SCI, thereby improving the outcome. These results indicated a beneficial effect of splenectomy within 6 h after SCI on either early or late outcomes after SCI, but surprisingly no notable improvement was seen in rats receiving splenectomy over 12 h after SCI. Therefore, the present study provides new insight to how splenectomy contributes to post-SCI neuroprotection. Our study is the first study to demonstrate that timing of splenectomy after acute SCI may differentially influence the outcome of SCI.

The spleen is one of the most important immune organs and contains a variety of immune cells. Splenectomy before stroke may decrease infarct volume through anti-inflammatory effect (Seifert et al., 2012; Zhang et al., 2013). In this study, we found that splenectomy in the *in vivo* SCI rat model significantly reduced glial activation if the spleen was surgically removed within 6 h after SCI. The histomorphometric change of the cord tissue sections in the splenectomy groups was further confirmed with NF200. Splenectomy within 6 h after SCI reduced the number of immune cells in the injured cord, specifically macrophages, neutrophils, B cells, and T cells. Therefore, splenectomy within 6 h after SCI has been shown to significantly reduce the number of infiltrating cells, and to improve functional recovery following SCI. Actually, the effect of splenectomy after SCI on the long-term outcome could possibly be explained by which splenectomy within 6 h after SCI significantly reduce cord inflammation and glial activation early after SCI.

FA data show a decrease in NonSPX group and SPX24 at days 14 and 28 after SCI, whereas there is significant increase in SPX0, SPX6, and SPX 12 groups. According to our previous report (Li et al., 2017), FA can be used to evaluate the severity of SCI at the early stage, that is to say, FA decreased with the presence of injury. In addition, we demonstrated that splenectomy within 6 h after SCI relieved the immune cell infiltration at day 3 after SCI. However, a direct relationship between FA and immune cell infiltration at different time points is not well evaluated. Therefore, whether the MRI data can be used to make inferences on immune cell infiltration is not clearly distinguishable.

In addition to the cellular responses in the injured cord to splenectomy, there are also changes in cytokine responses. Our data demonstrated that splenectomy within 6 h after SCI reduced the amount of IL-1β and TNF-α in the injured cord. This suggests that splenectomy within 6 h after SCI reduced the IL-1β and TNF-α levels locally, indicating that the spleen is a partial source of these cytokines after SCI and they aggravate the degree of SCI. Additionally, at 12 h after SCI, the splenectomized rats had just the same number of proinflammatory cytokines in the injured cord as the rats with intact spleens did. Since splenectomy did not have an effect on anti-inflammatory cytokine IL-10, it appears that partial anti-inflammatory cytokines do not play a significant role in neurologic outcome in SCI. These results implied that after SCI the spleen might release stored immune cells and proinflammatory cytokines into injured cord and promote secondary SCI.

This study is primarily limited to the histopathology of injured cords, thus providing partial insight into the pathophysiological changes in SCI. Our assessment is focused on splenectomy, but it is likely that multiple mechanisms could be involved in the neuroprotective effects of SCI.

Splenectomy is a double-edged sword in SCI. It can be both protective and deleterious, depending on when it is performed. The present study demonstrates that the inflammatory responses of SCI can be decreased by immediate splenectomy, to be exact, within 6 h after SCI, while delayed splenectomy (over 12 h after SCI) has no markedly neurologic protective effect. In other words, removing the spleen within 6 h after injury may be a clinical therapeutic management for SCI, but splenectomy over 12 h after injury is no longer necessary. Because the splenectomy is an invasive procedure, the risk of such surgical procedure would present a major obstacle to translating this method to clinical management of SCI.

In conclusion, the present study demonstrates that splenectomy is neuroprotective for SCI if performed at the right time. Early splenectomy (within 6 h or less after SCI) is associated with improved outcome, while the outcome with late splenectomy (12 h or more later after SCI) is not notably different from that without splenectomy. Based on these results, the present study suggests a potential novel strategy for spinal cord protection in the future. Currently, it is yet not practical to perform a splenectomy shortly after SCI in a human subject, but improving neurologic outcome by splenectomy might be warranted, when possible.

## Synthesis

Reviewing Editor: Eran Perlson, Tel Aviv University Decisions are customarily a result of the Reviewing Editor and the peer reviewers coming together and discussing their recommendations until a consensus is reached. When revisions are invited, a fact-based synthesis statement explaining their decision and outlining what is needed to prepare a revision will be listed below. The following reviewer(s) agreed to reveal their identity: Jae Lee.

The overall goal of this study was to investigate the effects of timing of splenectomy after SCI in rat contusion injury. Previous studies have shown that splenectomy leads to reduced leukocyte infiltration after SCI, which is associated with functional and histological benefits. However, most of these studies involved splenectomy prior to injury, so determining the therapeutic time window for splenectomy can provide useful information. By performing splenectomy at 0 (immediately), 6, 12 and 24 hours after SCI, the authors report that the first six hours is the time window for realizing the benefits of splenectomy. Although this is highly specialized information, it is very useful and important information to those in the field because in addition to splenectomy time window, it provides crucial insight into the time window for targeting leukocyte infiltration after SCI. However, there are major comments that must be addressed prior to publication.

Major Comments:

1) The longitudinal MRI/DTI data could be very useful since it can show potential correlations between MRI signal and immune cell infiltration, but the MRI data and tractography data need to be quantified. Also, the FA data shows a decrease in NonSPX group, which could account for the significant differences compared to SPX groups rather than an increase in the latter. These data should be discussed in the context of immune cell infiltration and whether the MRI data can be used to make inferences on immune cell infiltration.

2) Please combine the immuno results into one section

3) immuno images and their associated bar graph should be in the same figure.

4) The image quality for GFAP and NF200 are very poor and do not reflect the descriptions in the narrative (all the images look similar to each other). Please replace with higher quality images that reflect the changes described in the text.

5) Incorrect stats were used. A two-way ANOVA should have been used for BBB. No stat test is reported for immuno quantifications.

Minor Comments:

1) Please provide a more detailed narrative on the type of locomotor recovery observed between groups

2) All acronyms should be spelled out under results headings

3) In Discussion, the authors state that the spleen is the major source of IL1B and TNF based on their data, but this is at 6 hours post injury whereas these cytokines reach peak express within the first few hours after injury. Other cell types such as microglia are know to produce these cytokines very highly, and without comparing them directly, this is an overstatement. Please revise.

4) In Discussion, authors state that splenectomy does not affect anti-inflammatory cytokines. But this is based on IL-10 alone, which is an overstatement. Please revise.

## Author Response

Comment 1: The longitudinal MRI/DTI data could be very useful since it can show potential correlations between MRI signal and immune cell infiltration, but the MRI data and tractography data need to be quantified. Also, the FA data shows a decrease in NonSPX group, which could account for the significant differences compared to SPX groups rather than an increase in the latter. These data should be discussed in the context of immune cell infiltration and whether the MRI data can be used to make inferences on immune cell infiltration.

Author Response: Thanks for your suggestion. These were added in DISCUSSION section.

Comment 2: Please combine the immuno results into one section

Author Response: I have accepted the suggestion.

Comment 3: immuno images and their associated bar graph should be in the same figure.

Author Response: I have accepted the suggestion. I combined figures.

Comment 4: The image quality for GFAP and NF200 are very poor and do not reflect the descriptions in the narrative (all the images look similar to each other). Please replace with higher quality images that reflect the changes described in the text.

Author Response: We improved our picture quality, thanks.

Comment 5: Incorrect stats were used. A two-way ANOVA should have been used for BBB. No stat test is reported for immuno quantifications.

Author Response: I have accepted the suggestion.

Comment 6: Please provide a more detailed narrative on the type of locomotor recovery observed between groups.

Author Response: I have accepted the suggestion.

Comment 7: All acronyms should be spelled out under results headings.

Author Response: I have accepted the suggestion.

Comment 8: In Discussion, the authors state that the spleen is the major source of IL1B and TNF based on their data, but this is at 6 hours post injury whereas these cytokines reach peak express within the first few hours after injury. Other cell types such as microglia are know to produce these cytokines very highly, and without comparing them directly, this is an overstatement. Please revise.

Author Response: I have accepted the suggestion.

Comment 9: In Discussion, authors state that splenectomy does not affect anti-inflammatory cytokines. But this is based on IL-10 alone, which is an overstatement. Please revise.

Author Response: I have accepted the suggestion.
